# Personal positioning of oncology patients in palliative care: a mixed-methods study

**DOI:** 10.1186/s12904-022-00916-5

**Published:** 2022-03-11

**Authors:** Hellen Luiza Meireles Silva, Pedro Henrique Martins Valério, Cristiano Roque Antunes Barreira, Fernanda Maris Peria

**Affiliations:** 1 Department of Medical Images, Hematology and Clinical Oncology, School of Medicine of Ribeirão Preto, University of São Paulo, São Paulo, Brazil; 2 Ribeirão Preto, Brazil; 3 School of Philosophy, Sciences and Letters of Ribeirão Preto, University of São Paulo, São Paulo, Brazil; 4 School of Physical Education and Sports of Ribeirão Preto, University of São Paulo, São Paulo, Brazil

**Keywords:** Palliative care, Oncology, Meaning of life, Phenomenology, Quality of life

## Abstract

**Background:**

Advanced oncological disease requires comprehensive health care, although attention is predominantly paid to the physical dimension of care. The consideration of personal positioning encompasses other dimensions of patients’ management of their illness, such as existential management and expanding forms of care. The objective of this study was to understand the personal positioning of cancer patients in palliative care.

**Methods:**

This was a cross-sectional study using the mixed convergent parallel method. The sample consisted of 71 cancer patients in palliative care, of whom 14 participated in the qualitative and quantitative portions and 57 participated in only the quantitative portion. Phenomenological interviews were performed, and qualitative and quantitative methods were used to collect meaning of life (PIL-Test), quality of life (EORTC QLQ C-30), anxiety and depression (HADS) and sociodemographic data. The interview results were analysed according to the principles of classical phenomenology, and the quantitative data were analysed using the generalized structural equations model.

**Results:**

The results showed that the patients turned to living, focusing on their possibilities and distancing themselves from the impact of the illness and the factuality of death, which the patients themselves associated with not succumbing to depression, a condition whose signs were exhibited by 21% of the sample. Sustaining this positioning required a tenacious fight, which feeds on sensitivity to life. Linked to this position was the belief in the continuation of life through religious faith, together with the patients’ realization of the meaning of their lives. In this same direction, there was a direct association between awareness of the meaning of life and increased scores on the functional scales (*p* <  0.01) and decreased scores for symptoms (*p* <  0.01), anxiety (*p* = 0.02) and depression (*p* < 0.01). The last element that emerged and structured this experience was the intense will to live and a sense of the value of life.

**Conclusions:**

Through the use of mixed methods, the present study recognized the existential positioning of cancer patients in palliative care. This understanding can aid in the realization of more comprehensive and meaningful treatment plans and can contribute to the goal of achieving humanization in this area of treatment.

**Supplementary Information:**

The online version contains supplementary material available at 10.1186/s12904-022-00916-5.

## Background

Experiencing a life-threatening disease affects the patient in her or her entirety and requires comprehensive health care [1, 2]. Although there are efforts dedicated to providing such existential care, the predominant focus is on the evaluation and management of physical symptoms, even among professionals specialized in palliative care [3–5]. The existential dimension specifically contemplates human attitudes, one of which is the ability to position oneself (e.g. a decision, acceptance or rejection of a thought or impulse), since the human being is ontologically free; despite being affected by the condition, are not determined by it, since they have freedom in their personal positioning [6].

Different positions can be identified in this condition, and one of them is the search for opportunities to understand the meanings of one’s life [7]. The exploration of the meaning of life in the health field includes the possibility of having a healthier existence [8, 9]. Specifically, in palliative care, an association between high scores of meaning of life and low levels of distress, anxiety and depression has been identified [10]. Meaning of life is also associated with higher quality of life, which is the main goal for cancer patients in palliative care [11, 12].

The concept of quality of life, like health-related concepts, is usually considered only in terms of the disease; measurement instruments are fundamentally related to biostatistics, psychometrics and economics, and there are no analyses of key non-measurable issues, such as the individual’s history, context and values [13]. Qualitative studies with a phenomenological approach have broadened the understanding of the experiences of advanced cancer patients, revealing the impactful experiences of this type of existentially threatening illness, the uncertainties that patients feel about disconnecting with life and their attitude of holding on to life with hope [14]. The orientation towards everyday life centred in the present and guided by an existential sense and the possibility of a cure or control of the disease through treatments or by religious means, help patients to deal with the disease, allowing them to consider the value of life and the hopeful possibility of its continuity [15–19].

Despite advances in the area of palliative care in oncology in terms of exploring health in its full dimension, the literature includes few quantitative studies of the construct of meaning of life according to the framework of logotherapy and existential analysis, a perspective that delineates and contributes to the research regarding this construct. Furthermore, there are few studies that consider the meaning of life and apply complex methodologies that are capable of simultaneously analysing several variables and possible confounding factors. In qualitative research with a phenomenological approach, some studies present possible methodological issues that can be advanced, such as the presence of theoretical assumptions in interview scripts that incline the participant’s statements to reflect what the researcher is researching, reducing research capacity; a focus on the disease or on coping without a more comprehensive description of the affected person’s experience; and, finally, deficiency in the in-depth descriptions of essential elements of how people live and the attitudes that they assume under the condition of an advanced disease—that is, how they position themselves and, especially, how their positioning is sustained—through a comprehensive understanding of their lived experience. Therefore, unlike the previously published phenomenological studies on the subject, the present study does not take a thematic approach to coping (as a strategy for managing the disease that assumes what it is like to be ill) or position the disease as the object of experience (a focus that directs and thematically centres the interviews); instead, it starts from the condition of illness and broadly explores what the results revealed as a unit: the perceptions and positioning experienced under the condition of advanced oncological illness. This initial design of the research object follows a preliminary phenomenological approach to data collection and analysis, as recommended by Barreira [20]. It is a matter of suspending the object of research as a topic in order to address it as an experience. Aligned with the philosophical anthropology inherent to logotherapy and existential analysis, this consideration of experience does not presume that it is determined by the condition imposed on the person; instead, it considers that the person’s perception and positioning are situated in relation to both the disease and other aspects of the patient’s life that are important to comprehensive health care. This openness to the totality of experience, as well as to its possible interrelationships, is what is designated in the present research as an existential dimension.

Given the above, the study uses in-depth reports to investigate the essential elements of the personal positioning of patients with advanced cancer and how they support each other; simultaneous, it aims to verify the hypothesis that meaning increases quality of life scores and reduce the symptoms of anxiety and depression. Thus, the objective of this study is to conduct a descriptive analysis, based on first-person reported experience, of the personal positioning of cancer patients in palliative care and its interface with meaning of life, quality of life and anxiety and depression symptoms through the complementarity of the mixed methodology. This approach is important and does not exist in the literature.

## Methods

### Participants and procedures

This was a cross-sectional study using the mixed convergent parallel method of Creswell and Clark [21]. We applied the Consolidated Criteria for Reporting Qualitative Research Studies (COREQ) [22]. The convenience sample consisted of 71 cancer patients in palliative care, of whom 14 participated in the qualitative and quantitative portions and 57 participated in only the quantitative portions. The patients were undergoing outpatient follow-up at the Clinical Oncology Services and Palliative Care Group of the Clinics Hospital, School of Medicine of Ribeirão Preto, University of São Paulo (HC-FMRP-USP). The first author is a psychologist at the latter of these services, and patients treated by her were not included in the study. The inclusion criteria for both the quantitative and qualitative portions of the study were as follows: patients older than 18 years; both sexes; diagnosis of malignant neoplasm confirmed by anatomopathological examination; malignant neoplasm of the hepatocarcinoma type diagnosed according to the specific criteria for this neoplasm (this information was collected from the medical records); awareness of the diagnosis of the palliative stage; any location as a primary site; stage IV updated at the time of recruitment for this cancer study; a Karnofsky Performance Scale (KPS) score greater than or equal to 50; and outpatient clinical follow-up by the team at the Clinical Oncology Service and/or Palliative Care Group of HC-FMRP-USP. The non-inclusion criteria were cancer patients undergoing curative cancer treatment, patients with noncancer diseases and those who were not seen by the first author. The exclusion criteria were patients with difficulties understanding/responding to the Consent Form and the questions contained in the instruments and the interview. All ethical principles for research involving human beings in Brazil were considered, and the Declaration of Helsinki was followed. The study was approved by the research ethics committee of the institution where it was conducted under Process HCRP No. 10402/2017.

The collection procedures were initiated through a triage of the institution’s electronic medical records, which offered the list of the day’s patients, and the inclusion and non-inclusion criteria were verified. Next, the patients were invited to participate in the study in the outpatient clinic waiting room. Those who agreed to participate were taken to the palliative care room, where the instruments were applied. Data collection began with the qualitative measures, and the 14 patients who participated received Informed Consent Form 1 (ICF1) to read and sign. To avoid fatiguing the patients, they returned on another day according to their availability to complete the quantitative measures. Fifty-seven participants completed the quantitative phase and received the Free and Informed Consent Form 2 (ICF2) to read and sign. After data collection was completed, the last author of this manuscript/article, a medical oncologist, confirmed that all patients were in the palliative stage of disease.

### Instruments

Physical performance was assessed using the Brazilian version of the KPS scale, which was developed to assess physical capacity in cancer patients [23]. The level of attribution of meaning in life was evaluated by the *Purpose in Life Test* (PIL-Test), which was originally created by Crumbaugh and Maholick [24] and adapted for Brazil first by Aquino [25], and then by Nobre [26]. The adapted Brazilian test contains 14 items with a Likert-type response scale from 1 to 7 points and a total score ranging from 14 to 98 points [26]. Quality of life was assessed using *European Organization for Research and Treatment for Cancer Quality of Life Questionnaire* (EORTC QLQ C-30), version 3.0. validated for Brazil [27]. The EORTC QLQ-C30 questionnaire has 30 questions related to five functional scales (physical, functional, emotional, social and cognitive), a scale related to the overall state of health/quality of life, three symptom scales (fatigue, pain and nausea/vomiting) and six additional symptom items (dyspnoea, insomnia, loss of appetite, constipation, diarrhoea and financial difficulties). Symptoms of anxiety and depression were screened with the *Hospital Anxiety and Depression Scale* (HADS), which was created by Zigmond and Snaith [28] and validated in Brazil by Botega et al. [29]. The established cut-off for outpatients is 8 points, and this parameter was used in the present study.

In the phenomenological interview, the patients were invited to narrate their lived experiences with the aim of achieving a mutual understanding by the participant and the researcher [30]. The interview was conducted by the first author and supervised by the second and third authors, who are experts conducting in this type of interview. The interview was open and in-depth, as recommended by Revan [31], and began with a contextualization question: “How did your illness begin?” After this question, two guiding questions were asked in a logical and graded sequence of complexity: “What is it like for you to go through the experience of illness?” and “How are you coping with this experience of becoming ill?” Based on the answers, other questions were asked to direct the patient in the thematized experience (the personal positioning within the condition of illness) to elucidate and deepen the responses. The empathic phenomenon, as elucidated by Edith Stein [32], leads the interviewer to understand the experience of alterity through suspensive listening. This concept describes an attitude and synthesis of intersubjective operations guided by empathy to accompany the reported experience, name it, clarify it and confirm it, when necessary, pursuing its meanings and lived experiences. For a long time, empirical phenomenological research used the interview as only a data collection instrument, restricting the phenomenological moment to the subsequent analysis of these data [20, 33]. The finding that this procedure was insufficient for an investigative design with an entirely phenomenological arc was concomitant with the finding that to analyse personal experiences, collecting opinions and personal considerations about events or facts is insufficient, as it is necessary to have an intersubjective production of reports of experiences as they are actually lived, in first person. Thus, suspensive listening was conceived [20, 30, 33] and applied [34, 35] as a methodological description and prescription of the attitude and operations necessary for an interview to be called phenomenological.^1^ In suspensive listening, the interviewer refrains from using theories, prejudgments, categorical frameworks and abstractions about a phenomenon to address the interviewee’s personally lived experience, filling in his or her reports of experience. This delimits the meaning of the interview questions [30]. As a result, the interviewer’s questions and attitude of suspension provoke the interviewee and ask him or her to abandon the role of objective informant for a personalized, confident telling of how he or she lived what he or she lived.^2^ The application of this approach requires a clear definition of the experience one wants to know and emphasizes attention to the respondent’s report during the interview. This process is characterized by an intersubjective dynamic in which the suspensive listening of the interviewer modifies the attitude of the interviewee.^3^ Each interview lasted approximately 60 min, was recorded with the consent of the participants and was fully transcribed by the first author for subsequent analysis. Sociodemographic data and clinical information contained in the medical records were also collected. All data were collected from native speakers of Portuguese.

### Statistical and qualitative analysis

The relationship of meaning of life with the EORTC QLQ-C30 domains was analysed using the multiple linear regression model, controlling for anxiety, depression, age group, gender, income, marital status, religion, education and cancer treatment (possible confounding factors). To simultaneously analyse the relationships among meaning of life, quality of life (overall, functional and symptom scores) and symptoms of anxiety and depression and possible confounding factors such as age group, gender, income, marital status, religion and cancer treatment, a generalized structural equation model (GSEM) was used (see Additional file 1)). The use of the GSEM allowed us to estimate relationships involving continuous and binary variables, and the binary variables were estimated using a probit model [38, 39]. The estimation method used was maximum likelihood (ML). The analyses were performed using STATA software version 14.0 [40]. For all comparisons, a significance level of 5% was adopted.

The qualitative data were analysed by removing preconceptions, judgements, theories and previous beliefs about the meaning of the phenomenon, according to Barreira [33]. The first step was psychological reduction, a process that individually analyses the reports produced from a personalist approach. This description is correlative to the “psychological subject”; that is, it approaches the personal experiences of each subject in a manner that is disconnected from any interpretive or conceptual judgement about the report. This delimits this analytical moment to the apprehension and description of significant events (in terms of first-person experiences rather than facts or rational information) related to the investigated experiential object of each patient separately. Then, intentional crossing was performed in which interviews were compared to identify invariant aspects of the lived experiences. Then, intersubjective reduction was performed, which deepened this crossing through successive eidetic or imaginary variations; this procedure is based on the phenomenology of Edmund Husserl, who stated that its purpose is to imaginatively alter the phenomenon in question to identify the elements without which the phenomenon would not be recognized as such [33, 37]. Following the mixed convergent parallel method, the results were produced separately and then compared, and their differences and similarities were observed and highlighted to extract their essential elements and achieve an interpretation of the phenomenon under study [21, 41].

## Results

### Quantitative results

**Fig. 1:**
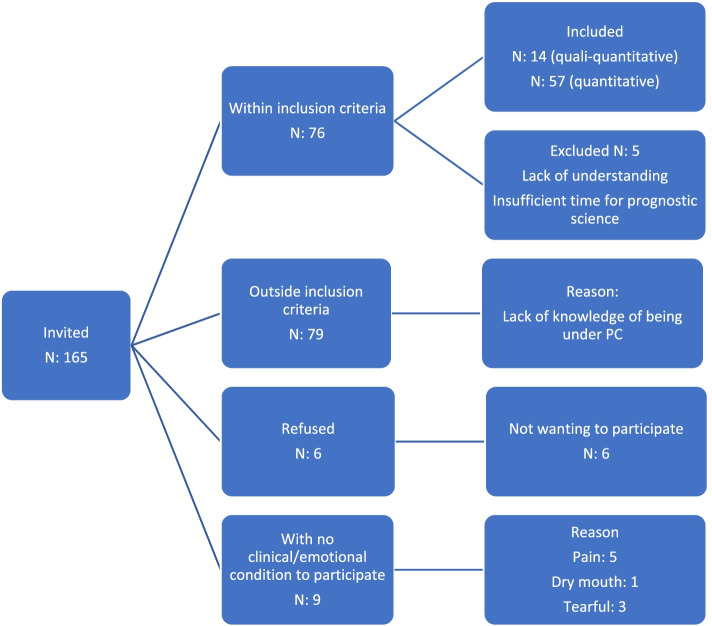
Organogram of the flow of data collection. PC = Palliative care

The numbers related to the collection process are presented in the organization chart shown in Fig. 1.

Regarding sociodemographic data, there was a predominance of female patients (58%) and patients who were younger than 60 years (55%), had partners (65%), had up to 8 years of education (55%), earned up to 4 minimum wages (84%) and were Catholic (66%). Regarding clinical issues, most had a KPS score of 80 to 100 (76%), and almost all received cancer treatment (91%). Regarding the mental and existential health measures, 21% presented depression, and 24% presented anxiety.

The GSEM showed the direct and indirect effects between meaning of life, quality of life, anxiety and depression symptoms, age group, gender, income, marital status, religion and treatment (see Additional file 2).

Table 1 below shows the direct effects among the variables included in the GSEM, and Table 2 shows the relevant indirect effects found in the model.

**Table 1:** Direct effects between the variables included in the GSEM

Variable	Anxiety		Depression		Global health status		Functional scales		Symptoms scales		Meaning of life	
	EC (CI)	*p*	EC (CI)	*P*	EC (CI)	*p*	EC (CI)	*p*	EC (CI)	*p*		
*Meaning of life*	−0.05 (− 0.09 to − 0.01)	0.02	− 0.08 (− 0.14 to − 0.02)	< 0.01	− 0.06 (− 0.41–0.30)	0.76	0.39 (0.12 to 0.66)	< 0.01	− 0.74 (− 1.05 to − 0.42)	< 0.01		
*Marital status* With partner vs. without partner	− 0.54 (− 1.52 to − 0.43)	0.28	1.04 (− 0.70 to 2.78)	0.24	0.53 (− 7.62 to 8.68)	0.90	0.90 (− 5.69 to 7.48)	0.79	1.62 (− 7.05° 10.30)	0.71	−5.87 (− 12.09 to 0.34)	0.06
*Income* Up to 4 sm vs. Above 4 sm	1.50 (− 0.44 to 3.45)	0.13	0.00 (− 2.37 to 2.38)	0.99	2.25 (− 8.68 to 13.19)	0.69	− 3.61 (− 12.41 to 5.18)	0.42	5.46 (− 6.07 to 17.00)	0.35	−0.84 (− 9.45 to 7.77)	0.85
*Religion* Catholic vs. other	−0.58 (− 1.96 to 0.79)	0.41	− 1.12 (− 3.86 to 1.63)	0.43	11.03 (− 1.35 to 23.41)	0.08	− 10.05 (− 19.78 to − 0.32)	0.04	−16.14 (− 28.41 to − 3.87)	0.01	−2.66 (− 11.62 to 6.31)	0.56
*Religion* Evangelical vs. other	− 0.52 (− 2.07 to 1.04)	0.51	− 0.85 (− 3.69 to 1.99)	0.56	11.29 (− 2.77 to 25.36)	0.12	−11.91 (− 22.94 to − 0.89)	0.03	− 8.43 (− 22.85 to 5.98)	0.25	−0.12 (− 10.57 to 10.33)	0.98
*Age range* < 60 vs. > 60	1.36 (0.19 to 2.52)	0.02	0.11 (− 1.28 to 1.50)	0.87							4.52 (−1.83 to 10.87)	0.16
*Treatment* Yes vs. no	0.32 (−1.80 to 2.43)	0.77	5.26 (Not estimated)	0.96	6.80 (−9.20 to 22.79)	0.41	22.79 (10.99 to 34.58)	< 0.01			−7.51 (−18.45 to 3.43)	0.18
*Gender* M vs. F	0.29 (−0.74 to 1.32)	0.58	0.76 (−0.67 to 2.20)	0.3	5.26 (−3.23 to 13.74)	0.23	6.74 (0.07 to 13.41)	0.04	−9.03 (−17.57 to −0.48)	0.04	2.13 (−4.24 to 8.50)	0.51
*Education* up to 8 years vs. over 8 years											−3.73 (−10.24 to 2.78)	0.26
*Global health status*	−0.04 (− 0.07 to − 0.01)	< 0.01	0.03 (− 0.01 to 0.07)	0.14								
*Functional scales*	0.00 (− 0.03 to 0.03)	0.93	− 0.10 (− 0.15 to − 0.02)	0.02	0.42 (0.13 to 0.71)	< 0.01						
*Symptoms scale*	0.01 (−0.02 to 0.04)	0.48	−0.06 (− 0.11 to 0.00)	0.06	0.02 (− 0.26 to 0.29)	0.92	− 0.59 (− 0.77 to − 0.41)	< 0.01				

*EC (CI)* Estimated coefficient (confidence interval)

**Table 2:** Relevant indirect effects found in the GSEM

Outcome	Independent variable	Mediating variable	Estimated coefficient	*P* value
Depression	QoL symptoms	Functional QoL	0.06 (0.01 to 0.11)	0.03
	Tto (Yes vs. No)	Functional QoL	−2.25 (−4.47 to − 0.02)	0.04
Global QoL	Tto (Yes vs. No)	Functional QoL	9.54 (1.33 to 17.74)	0.02
	QoL symptoms	Functional QoL	−0.25 (− 0.43 to − 0.06)	< 0.01
	Meaning of life	Functional QoL	0.16 (0 to 0.32)	0.04
Functional QoL	Gender (M vs. F)	QoL symptoms	5.34 (0.04 to 10.63)	0.04
	Religion (Catholic vs. other)	QoL symptoms	9.54 (1.75 to 17.33)	0.02

*QoL* Quality of life, *Tto* Treatment

In terms of the direct effect, meaning of life was related to increased functional scale scores (*p* < 0.01), decreased symptom scores (*p* < 0.01) and decreased symptoms of anxiety (*p* = 0.02) and depression (*p* < 0.01). Additionally, indirectly, meaning of life was related to an increase in global health scores mediated by the functional scale scores (*p* = 0.04). No sociodemographic variables (gender, age, education, income, marital status or religion) or treatment had a direct association with meaning of life, i.e., the patients’ general living conditions and treatment status were not related to high or low meaning of life scores. Gender was directly associated with the functional scale and the symptoms scale scores, with men showing higher scores on the functional scale (*p* = 0.04) and lower scores on the symptoms scale than women (*p* = 0.04). Gender was indirectly associated with the data from the functional scale moderated by the symptom scale (*p* = 0.04), with men showing fewer symptoms and, consequently, higher scores on the functional scales. Age was directly related to anxiety, i.e., patients younger than 60 years (*p* = 0.02) had a mean score of 1.36 points higher on the scale than the elderly. Religion was directly and indirectly associated with the functional scale score and directly associated with the symptoms scale score. The Catholic (*p* = 0.04) and Evangelical (*p* = 0.03) religions were associated with decreased scores on the functional scales compared to other religions (Buddhism, Candomblé, Spiritism and Christianity). The Catholic religion was only associated with increased functional scale scores through the mediation of the symptom scale score (*p* = 0.02) because Catholics had lower symptom scores and, consequently, better scores on the functional scales. The provision of palliative cancer treatment also had a direct effect on the functional scale scores (*p* < 0.01), significantly increasing them (to a mean of 22.79 points), and had an indirect effect on overall health status (*p* = 0.02) and depression (*p* = 0.04) via the functional scale scores. This means that palliative treatment increased functional scale scores (including physical function, role performance and social function) and, consequently, increased overall health status and decreased the depression scores. Overall health status had a direct effect on anxiety (*p* < 0.01) because it decreased the occurrence of anxiety symptoms. The functional scales had a direct effect on overall health status (*p* < 0.01), increasing its score, and on depression symptoms (*p* = 0.02), decreasing their score. The symptoms scale score had a direct decreasing effect on the functional scale score (*p* < 0.01) and had an indirect effect on the overall health status (*p* < 0.01) and on depression symptoms (*p* = 0.03) via the mediation of the functional scale score. These relationships indicate that the symptoms scale score decreased the functional scale scores, which consequently decreased the overall health status score and increased the depression symptoms score.

### Qualitative results

The qualitative results obtained using the phenomenological orientation revealed the experience of becoming ill with a palliative oncological conditions in the participants’ daily lives. These results represent an apprehension of the experience in its entirety, identifying the experiential elements that structure it and are intertwined with one another, configuring the motivational aspect of existential positioning that can be called the fight for life. This description is compatible with the model of phenomenological psychology in the works of Edith Stein [32] and Edmund Husserl [37] and can be observed in the participant accounts presented below:

*(...) this is what I say, not putting everything on your head, because I think it gets in the way if people focus on it, they keep it in their head every day. There are days when I don’t even think I have this kind of thing, you know? (...) So you have to forget that you have the problem and move on with life, work, and go on living.* (Patient 12)

It was observed that the patients avoided thinking about their incurable disease and focused on other aspects of their lives in a deliberate effort to avoid such thoughts in daily life, as evidenced by the patients’ accounts of the consequences of this position:

*"When you start to experience depression, you know, it gives me that agony, that fatigue afterwards... so I forget because I think we should not worry too much (...) to put negative things, disease problems in the head, thinking about it, that it will get worse, that it will do this, that it will happen, because the worst thing that exists is this depression (...) I deviate from thinking [about it], right?* (Patient 13)

The thematization of incurable illness involves the worsening of the physical condition, in the near or distant future, followed by death. The patients constantly strived not to focus on this subject in a deliberate effort to avoid the negative psychological impact of such thinking. The issue of temporality presented itself decisively here because the patients lived their daily lives and their possibilities in the present time, avoiding the psychological suffering caused by anticipating future difficulties inherent to having incurable cancer. This did not mean that they did not discuss illness and finitude; rather, they avoided letting themselves be attached to these issues at all times in their daily lives. More precisely, they presented a type of relationship with life that was based its possibilities and on restriction and loss: “I’m fighting... I’m fighting, and I’m not going to give up. I will not give up. I come to the doctors; I do everything right... because I want to live longer (...).” (Patient 3) It is a deliberate personal position of a fight against what decreases life under the new existential condition in favour of what could prevail as a realization under this same condition.

This fight, inscribed in the lived condition of the disease, highlights the possibilities of living. The condition of illness itself changed the way the individual perceived life, mobilizing an affective and evaluative sensitivity, though not an aesthetic one, towards the world and others, which, when noticed, nourished and contributed to a positioning that the patients sought to sustain and renew. From this point of view, if on the one hand, there is a motivation to live, on the other, there is living even when it exerts an attractive force. These are personal findings and discoveries that are appreciated as a new quality, capable of sustaining a person’s motivation to live: *“So I discovered that I really was loved. I knew they liked me, but only that we don’t know the dimension of that love that the person has for you*” (Patient 4).

This sensitivity is shown, therefore, as a sustained motivation between what life can offer at each moment of the here and now and what, in those who project a hopeful vision, could happen in the future as a fulfilment and continuation of living. A trait of surrender was observed that permeated this positioning: a surrendering of oneself to the here and now of everyday life in which one does not seek to know, but rather, to live and do: “*So, well... I don’t know how my end will be. (...) So I said, ‘Look, I will put myself in the hands of God’ (...), so I just ask God to give me quality of life, and I have it”.* (Patient 1) There is a surrender to the present moment, inseparable from the declared surrender the possibilities of continuing life and not to impossibilities. In this structural element of the positioning that is based in the meaning of life and enhanced by illness, there is recognition of different ways of surrendering to something that exceeds what can be understood, predicted or known, exhibiting an existential religious orientation in which the person and his or her own life are given over to something greater that, somehow, could decide their fate in life and death. What the reports showed is that this experiential element intensifies the value of the here and now of life: “*I only ask God to give me quality of life, and I have it*”. The relationship of care and trust in the divine has as a correlate care for oneself and the indeterminacy of the horizon predetermined by the disease. In this way, God emerges as a sense that invigorates, sensitizes and strengthens the person in his or her bond with the present, with life and with the divine. Thus, a belief in the continuity of life appears in a significant way, presented as an intimate connection with religious faith, with aspects of security and hope, that opens a horizon of possibilities for achievements in the present and the future: *“I think that faith helps us not to despair anymore, better to say so, to have hope, to have hope for the future, to live longer (get emotional)”.* (Patient 10) The experience of the continuity of life and its value is also embodied in the attentive and affectionate presence of the medical team and close friends and family, who, together with the patient, do not focus on the thematization of illness and/or believe in the continuity of life: “*Without help, we cannot cope with winning, the doctors, the friends, the family (...). Oh... I will go far, God willing.”* (Patient 9).

The continuity of life is strengthened in the relationship with what, in the here and now, is revealed as the meaning and fulfilment of life: *“(...) because I want to live more, you know? A little more; see my children; my daughter is getting married next year; I have two grandchildren. So I want to live a little longer (....).”* (Patient 3). There is, therefore, feedback between faith in the continuity of life—faith in God, the enjoyment of the present and the motivation/reason for living. Invariably, this experience is expressed more intensely when patients relate it to significant people: “(...) *they [are] there.... so I think it gave me the strength to say, ‘Why am I going to give myself up if I have my children, who are giving me strength? They still need me’”.* (Patient 1) Thus, the meaning of life and its value emerge, strengthening the patient’s efforts to live their existence.

## Discussion

It is not uncommon for health professionals, when faced with the positions that the participants described, to be perplexed, as they are dealing with patients whose lives are threatened by incurable cancer [42]. Such responses points to the relevance of better understanding the experience of this type of illness and expanding the reflections and practices of palliative care in oncology on the basis of this understanding. A focus on living was also identified in other studies, indicating aspects of connecting with life in the present through adaptation strategies, including focusing on the positive aspects of life and activities that are yet to be performed [14, 15, 43, 44]. However, most of the consulted studies do not clearly show how these elements articulate and support one another; in contrast, the present study evidences this aspect as a result of the way in which the phenomenological approach was used. The deliberate avoidance of thoughts and speeches about the incurable nature of the disease was one element of personal positioning, an attitude that is sustained by deepening the bond with life and possibilities yet to be accomplished; efforts are made to avoid damaging this attitude by being attached to limitations and to the threat of clinical worsening followed by death. The study by Arantzamendi et al. [43] confirms this finding by pointing to patients’ deliberate control of thoughts related to the disease and death to allow themselves to live in the present moment, and the present study’s contributions support this deliberate positioning. The study adds that patients perceive that thoughts about the disease are not useful to them, and the researchers perceived that when such thoughts are intrusive and persistent, conflicts and feelings of anxiety, fear and anger return, hindering the process of accepting the disease. The patients in our study were aware of these consequences and indicated their fear of having some mental disorder, such as depression. This perception is associated with data indicating that depression is the most prevalent symptom in palliative care patients [45], and it was present in 21% of our sample. It has been verified that disease recurrence and the confrontation of finitude may generate intense and prolonged emotions [46–48]. Anxiety was most common in patients younger than 60 years, a finding corroborated by the study by O’Connor, White, Kristjanson, Wilkes [49], but these data do not seem to have a consensus in the literature, which presents very different results [50, 51]. This avoidance of thoughts about illness and death, sustained by a bond with the possibilities of living, can help patients to recognize and manage their personal resources, enable them to distance themselves from the situation and direct them towards possibilities that are yet to be realized [6, 44].

It was identified that this positioning of avoiding daily contact with the condition of having incurable cancer is based on their fights. Other studies observed the occurrence of such deliberate efforts, noting that they required great energy, determination and resistance [15, 18, 42, 44]. The act of fighting shows the “power of resistance of the spirit” in the human being, since it is not determined by the individual’s numerous and rigorous conditions and is therefore able to shape his or her way of acting [6]. The fight of our patients takes specific forms, and one of them is the fight to perform tasks/responsibilities, which was also identified in the study by Willig and Wirth [14]. Other studies have considered tasks as only an orientation in life [15, 43] or as actions that are incompatible with the disease condition and are used to escape reality [42]. The results of the present study show that the latter interpretation is hasty and phenomenologically questionable, since patients who are anchored in the meaning of life are aware of the threat that they face and engage in tasks that correspond intimately with their daily reality—past, present and future. In this same direction, the literature indicates that participating in everyday life has a powerful positive influence on coping with the disease, enabling normality, identity and adaptation to changes [15, 44, 52]. In the same vein, our quantitative study showed that patients with better functioning as a result of oncological treatment showed a decrease in depressive symptoms. Generally, studies report the relationship between these variables in the opposite direction, i.e., that lower rates of depression are associated with higher quality of life [53, 54]. However, the relationship reported in the present study also occurs, since patients with better functioning can pursue tasks that have pleasure/happiness as a side effect [6], which helps to reduce depression. Further studies on the relationship between quality of life and depression are suggested.

The sensitization towards life was identified in this study to condition and nourish the will to fight, allowing patients to more clearly perceive their possibilities for action and their affections, the aesthetic face of the world around them and the value of life. The literature presents something similar in the intensification of experiences of lived moments as a result of the connection with the present time [14, 15, 44]. Our study adds that sensitivity to life is part of this process, showing how patients live in the present moment, more strongly recognising the quality of each experience, gathering its most significant and fundamental elements and, in this way, organizing the conditions for living intensely the present, as the literature describes.

Another element of the patients’ lived experience that was found in this study was having a reason for living, even amid uncertainties. Patients with advanced cancer may have deep insights regarding the essential goals of life [55], reflecting the human capacity to search for and find meaning in life and suffering [6, 8]. This search for meaning is the characteristic motivation of the human being; it dynamically appeals to the individual’s most important aspirations, and, when pursued, makes life happier and healthier [9, 56–58]. The study by Haug et al. [15] identified that meaning was central to the experiences of their elderly sample, helping them cope with the declines and losses arising from disease and protecting them from some age-related difficulties. The quantitative survey conducted in the present study verified the positive relationship between the meaning of life, increased quality of life and decreased in anxiety and depression, corroborating the literature on the subject [10–12]. The meaning of life is also related to a decrease in physical symptoms, which is a new finding in the literature and is relevant because one of the principles of palliative care is the rigorous control of physical symptoms in the effort to achieve quality of life [1, 2]. The study by Pontes [58] of patients with HIV indicated a similar finding: that the meaning of life was correlated with improved affections, which in turn was associated with the improvement of CD4+/CD8 + T lymphocyte indices. According to the theory of logotherapy and existential analysis, this relationship is explained by the realization of meaning, which has the side effect of pleasure/happiness, a concomitant and unintentional phenomenon, on psychophysical levels [6, 57, 58]. However, more studies are needed in this area to clarify and prove this relationship. Another new finding in the present study is that the meaning of life or its loss is not related to patient sociodemographic variables, which was also found by Dobríková et al. [11] with Slovenian patients. These results are consistent with the anthropology of Frankl [6, 57], who believes that the will to find meaning in life is ontological. However, further studies in other cultures are needed.

The meaning of life appeared to be imbued by an intense will to continue living, which was articulated in terms of beliefs of a religious nature when the patients attributed the possibilities of living to the divine, thus creating a horizon of hope. The phenomenology of religious experience designates it as contact with a power that surpasses and sustains the subject who contacts it [59]. A similar finding was reported in the Brazilian literature [16, 60–62] and in some foreign studies of a population with a religious tradition [18, 63]. In patients from other countries who did not have a religious belief, as in most studies in Europe and North America, there was a hopeful belief in the continuity of life being supported by medical treatments that had been performed or were still available [14, 15, 43]. Hope favours patients remaining active and engaged in life, even in extreme contexts, and is a central element of the resilience process [15, 17, 55, 64]. In the present study, belief in the continuity of life was embodied in the attentive and affectionate presence of the medical team and close family and friends who, together with the patient, do not discuss the disease, sharing beliefs similar to those of the patient or empathetically following the patient’s direction. Affective relationships with people who demonstrate empathy and a desire to spend time with the patient help keep hope alive [12, 15, 17, 65]. The significance of the health team being aligned with the patient and following his or her direction, paying attention to his or her real needs, has been emphasized [66]. The quantitative results of the present study revealed different associations among religions for one of the quality of life scales, but the greater number of Catholics (*n* = 47) and Evangelicals (*n* = 14) may have biased the data. There are no similar findings in the literature regarding differing effects on quality of life among religions, and future studies are needed to clarify this issue. Nonetheless, the findings emphasize the therapeutic capacity for hope through faith in palliative care, a stance that challenges interpretations that assume that such positioning represents denial or escape from reality and deprecates faith as a means of seeking the continuation of life or a change in one’s life situation, as noted in the Brazilian study by Reis, Farias and Quintana [67].

Linked to the belief in the continuity of life through religious faith was the will to live, which appeared as another element associated with valuing life. This desire to live was also noted in the study of Neirop-Van Ballen [19] as the primordial source of hope. In turn, in the study by Benites, Neme and Santos [16], patients directly reported valuing life and finding new meaning in it. Both studies corroborate the findings of the present study and further advance the assertion that valuing life is an element that structures patients’ positioning and is related to a religious belief in the continuity and realization of life. The will to live and the value of life are not clearly stated in most studies, but their existence is implied by statements that patients hold on to life and wish to live it with all of its intensity [14, 15, 44]. These experiences are similar to the worldview of Frankl [6], who affirms life and its unconditional meaning in any circumstance.

The number of patients included in the quantitative portion of the study did not allow for a randomized design. The number of subjects was limited by the exclusion of the 51% of prospective participants who were unaware of their prognosis, which was an inclusion criterion. Regarding religious affiliation, there were no atheists or agnostics in our sample, which could modify or expand the results regarding faith in the continuity of life or the way of dealing with illness and death in Brazilian patients. Therefore, further studies that include these populations are needed. Although the present quantitative study identified statistically significant direct and indirect associations among the variables, for the most part, it did not show clinical relevance when the levels of reliability and confidence intervals were considered. This may have occurred due to the use of the PIL-T because it was difficult to understand and patients experienced discomfort when answering it. It is suggested that future studies investigate the use of different instruments to evaluate the construct of meaning of life and its relationship with the variables used in this study.

## Conclusions

The patients who participated in the qualitative part of the present study were focused on the possibilities of living in the present time and extending life into the future, however indeterminate and short-term. The intensification and relative distancing of the present are correlates of distancing from the disease condition and the threat of death, and the meaning and value of life, accompanied by the hope of continued life through faith, standing out. This description shows an investigation based on phenomenological proposals that allowed a new and more precise explanation of how the elements are sustained and constituted the complex phenomenon of the human positioning of patients with advanced cancer, thereby contributing to the field of qualitative phenomenological research and to the fields of oncology and palliative care.

Consistent with these reports, the quantitative survey found that a sense of the meaning of life had a positive impact on the quality of life and mental health of the patients in the present study, indicating a position of hope and an assertive appreciation of life. New data to be investigated in the future were also identified, including the relationship between the meaning of life and a reduction in physical symptoms and the absence of a relationship between the scores on the meaning of life construct and the sociodemographic variables included in the study.

It is hoped that these results will prompt health professionals in oncology palliative care to a adopt broader and more comprehensive positioning of the human person under these circumstances, observing how they live, especially the meanings of their distance from the disease and their search for meaning in life, their efforts to live the personal values that are threatened by the disease and what sustains such efforts. Such efforts will make allow better monitoring of patients that start not from preconceived assumptions that they are denying or not understanding the disease, but from a recognition that in the patient’s experience, it is helpful to be guided by his or her own senses. In this way, this research can serve as another aid in achieving the goal of humanization and quality of life in palliative care in oncology.

## Supplementary Information

**Additional file 1.** Structure of the GSEM. Global QoL represents the global health status scale, Functional QoL represents the functional scales and Symptom QoL represents the symptoms scales. All scales are part of the EORTC-QLQ-C30.

**Additional file 2.** Results of the GSEM. *Continuous lines = direct effect. Dotted lines = indirect effect. ** Global QoL represents the global health status scale, Functional QoL represents the functional scales, and Symptom QoL represents the symptoms scales. All scales are part of the EORTC-QLQ-C30.

## Data Availability

The quantitative datasets used and/or analysed in this study are available from the corresponding author on reasonable request. The qualitative datasets generated and/or analysed in the study are not publicly available due to the need to protect patients’ identities but are available from the corresponding author on reasonable request.

## Abbreviations

COREQ: Consolidated Criteria for Reporting Qualitative Research
EORTC QLQ-C-30: European Organization for Research and Treatment for Cancer Quality of Life Questionnaire
HADS: Hospital Anxiety and Depression Scale
HC-FMRP-USP: Hospital das Clinicas, Ribeirão Preto Medical School, University of São Paulo
GSEM: Generalized structural equation model
KPS: Karnofsky Performance Scale
PIL-T: Purpose in Life Test

## Footnotes

1 For this purpose, according to Angela Ales Bello [36], it is decisive to resort to the thoughts of Edith Stein and Edmund Husserl. The analysis of Edith Stein’s empathy supports the possibility of verifying and following the experience of another. In turn, Husserl [37], referring to the development of a perspective of psychology as a science related to the human person and his or her lived world, describes a personal orientation, which differs from a natural orientation (directed towards facts) and from a phenomenological orientation (reflexively directed towards essences). The personal (or personalist) attitude refers to the way someone personally engages and relates to what he or she lives, designating a layer of intentional experiences that always expresses the coalition between the subject and object of experience.

2 To this end, the “why” and the “for what” are abandoned in the formulation of the questions, which are always based on the question “how”.

3 This modification allows the deepening and exploration of the subject’s experience, breaking through the layers of factuality and rationalization that tend to regard interviews as data collection instruments.
