# Supplementary material for: Personal positioning of oncology patients in palliative care: a mixed-methods study
Source: BMC Palliat Care. 2022 Mar 11;21:34. doi: 10.1186/s12904-022-00916-5 (PMC8917691; doi:10.1186/s12904-022-00916-5)
Supplement: Supplementary file 1 — Additional file 1. Structure of the GSEM. Global QoL represents the global health status scale, Functional QoL represents the functional scales and Symptom QoL represents the symptoms scales. All scales are part of the EORTC-QLQ-C30. [file 12904_2022_916_MOESM1_ESM.docx]

**ADDITIONAL FILE 1**


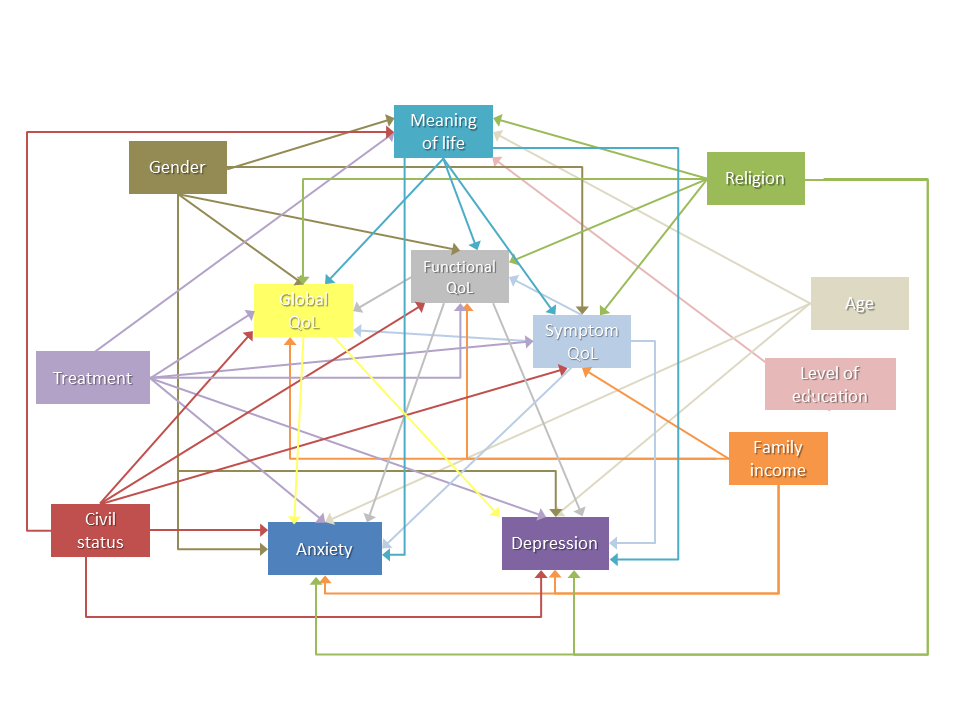


**Additional file 1.** Structure of the GSEM

Global QoL represents the global health status scale, Functional QoL represents the functional scales and Symptom QoL represents the symptoms scales. All scales are part of the EORTC-QLQ-C30.
