# Supplementary material for: Personal positioning of oncology patients in palliative care: a mixed-methods study
Source: BMC Palliat Care. 2022 Mar 11;21:34. doi: 10.1186/s12904-022-00916-5 (PMC8917691; doi:10.1186/s12904-022-00916-5)
Supplement: Supplementary file 2 — Additional file 2. Results of the GSEM. *Continuous lines = direct effect. Dotted lines = indirect effect. ** Global QoL represents the global health status scale, Functional QoL represents the functional scales, and Symptom QoL represents the symptoms scales. All scales are part of the EORTC-QLQ-C30. [file 12904_2022_916_MOESM2_ESM.docx]

**ADDITIONAL FILE 2**


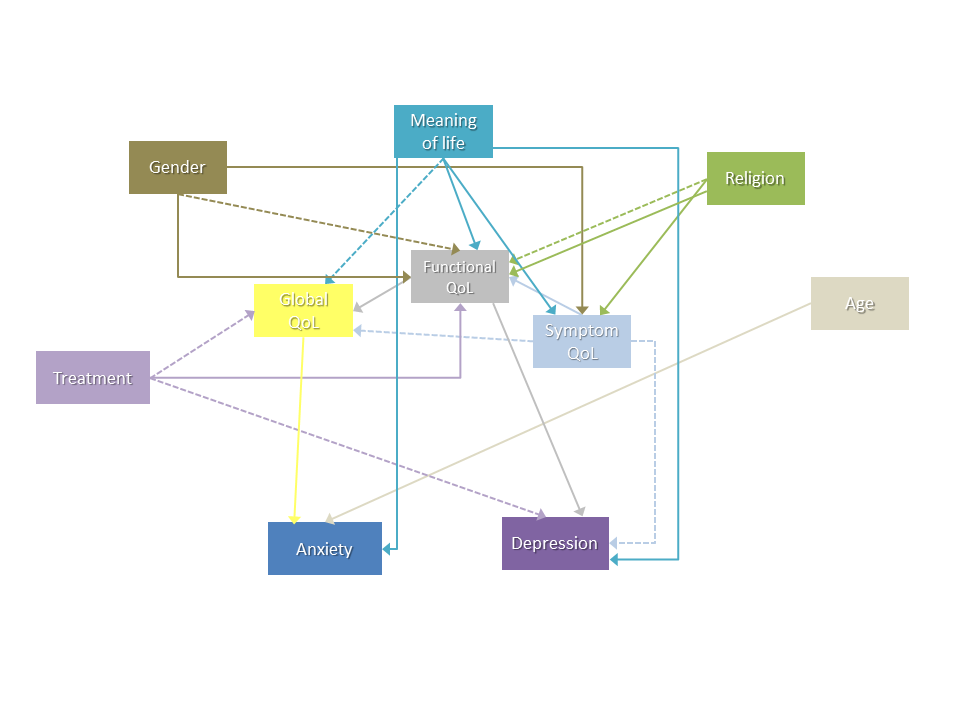


**Additional file 2.** Results of the GSEM. *Continuous lines = direct effect. Dotted lines = indirect effect. ** Global QoL represents the global health status scale, Functional QoL represents the functional scales, and Symptom QoL represents the symptoms scales. All scales are part of the EORTC-QLQ-C30.
